# Efficacy and safety of anlotinib plus programmed death-1 blockade versus anlotinib monotherapy as second or further-line treatment in advanced esophageal squamous cell carcinoma: A retrospective study

**DOI:** 10.3389/fonc.2022.942678

**Published:** 2022-08-17

**Authors:** Ying Liu, Qingqing Ge, Shuning Xu, Ke Li, Ying Liu

**Affiliations:** Department of Oncology, The Affiliated Cancer Hospital of Zhengzhou University & Henan Cancer Hospital, Zhengzhou, China

**Keywords:** anlotinib, programmed death-1 blockade (anti-PD-1), esophageal squamous cell carcinoma (ESCC), tumor immune microenvironment (TIME), efficacy and safety

## Abstract

**Background:**

Both anlotinib and programmed death-1 (PD-1) blockade have been approved for the second-line treatment of metastatic esophageal squamous cell carcinoma (ESCC). However, the combination of these two therapies has not been evaluated. This study investigated the efficacy and safety of anlotinib, a novel multitarget tyrosine kinase inhibitor targeting tumor angiogenesis, combined with PD-1 blockade as second or further-line treatment for advanced ESCC.

**Methods:**

Between January 2019 and February 2021, 98 advanced ESCC patients receiving anlotinib plus PD-1 blockade or anlotinib monotherapy as second or further-line treatment at Henan Cancer Hospital were retrospectively analyzed. Patients receiving anlotinib plus PD-1 blockade were grouped as cohort A (n=48), while those receiving anlotinib monotherapy were grouped as cohort B (n=50). The primary endpoint was progression-free survival (PFS). Secondary endpoints included the objective response rate (ORR), disease control rate (DCR) and toxicity. Furthermore, independent prognostic factors were identified by Cox regression analysis. A two-sided p-value of <0.05 was considered statistically significant.

**Results:**

Data was collected until May 1, 2021, with a median follow-up time of 9.30 months (8.23–10.37 months) in cohort A and11.10months (7.82–14.38 months) in cohort B. For patients with advanced ESCC, cohort A resulted in significantly longer PFS (5.40 vs. 3.00 months, P<0.001) and higher DCR (71.7% vs. 47.9%, P=0.019) than cohort B. The ORR indicated no significant difference between cohort A (23.9%) and cohort B (10.4%) (P=0.082). Adverse reactions were mainly grade1/2 in the two groups. Compared with cohort B, a significantly higher rate of grade 1–2 hypothyroidism was observed in patients in cohort A (P= 0.034). Three patients (6.3%) developed grade 1/2 immune-related pneumonia. There was no significant difference in the incidence of grade 3-4 toxicities. Multivariable Cox regression analysis showed that the drug regimen (P<0.001), Eastern Cooperative Oncology Group Performance Status (P=0.002), distant organ metastasis (P=0.008), and metastatic sites (P=0.032) were independent prognostic factors for PFS.

**Conclusions:**

Anlotinib plus PD-1 blockade showed promising anti-tumor activity and manageable toxicity as second or further-line treatment of advanced ESCC.

## Introduction

Esophageal cancer (EC) is one of the most prevalent malignant tumors of the digestive tract and occurs in the epithelium of the esophageal mucosa. According to global cancer statistics in 2020, the morbidity of EC ranks 7^th^ and the mortality ranks 6^th^ among all malignant neoplasms worldwide (1). The two major histological types of EC are esophageal squamous cell carcinoma (ESCC) and esophageal adenocarcinoma (EAC). Approximately 70% of esophageal cancer cases worldwide occur in China, and 90% of these cases are ESCC (2). Most ESCC patients are diagnosed at an advanced stage with lymph node or distant organ metastasis at their initial visits due to the disease’s insidious onset and strong invasiveness, leading to poor prognosis (3). The 5-year survival rate of patients with advanced ESCC is less than 20% (4).

Over the past decade, platinum-based combination chemotherapy has been recommended as the standard first-line therapy for patients with advanced or metastatic ESCC in China (5–7). In the last three years, nivolumab, camrelizumab and pembrolizumab have been evaluated in combination with chemotherapy as first-line treatment for advanced ESCC in the CheckMate 648, ESCORT-1st and KEYNOTE-590 clinical trials, respectively. The trials revealed promising clinical activity and tolerable adverse reactions (8–10). However, subsequent treatment lines for advanced ESCC patients who failed the standard first-line regimen remain limited. In recent years, targeted anti-angiogenic drugs have attracted great attention. Pathological angiogenesis is an important factor in the proliferation and metastasis of neoplasms. The process of tumor angiogenesis is regulated by many factors, and vascular endothelial growth factor (VEGF) is one of the most critical mediators. The latter increases vascular permeability, promotes the proliferation and differentiation of vascular endothelial cells, and induces tumor angiogenesis by binding with the vascular endothelial growth factor receptor (VEGFR) (11). Furthermore, VEGF can reprogram the immunosuppressive microenvironment by inhibiting the maturation of dendritic cells, enhancing the expression of inhibitory checkpoints (PD-1, cytotoxic T-lymphocyte antigen-4 and lymphocyte-activation gene-3) in CD8+ T cells. It also increases the numbers of immunosuppressive cells such as myeloid-derived suppressor cells, regulatory T cells and M2-like tumor-associated macrophages. Studies have shown that the VEGF overexpression in ESCC is about 24%-74%. Co-expression of VEGFR1/2/3 and platelet-derived growth factor receptor (PDGFR α/β) at the transcriptional level were also discovered in ESCC. Therefore, blocking angiogenesis may be a strategy to inhibit tumor growth and metastasis (12, 13). Anlotinib is a novel multi-target small molecule tyrosine kinase inhibitor (TKI), which exerts an inhibitory effect against angiogenesis and tumor growth by blocking vascular endothelial growth factor receptor(VEGFR), platelet-derived growth factor receptor (PDGFR), fibroblast growth factor receptor (FGFR) and c-Kit. A multicenter, double-blind, randomized phase 2 clinical trial (ALTER-1102) was performed on patients with advanced ESCC who progressed after platinum-or taxane- based chemotherapy. The study showed that the anlotinib group had a significantly longer PFS (3.02 vs 1.41 months; P<0.001) and a higher DCR (64% vs 18%; P<0.001) compared with the placebo group (14).

The randomized phase III trials (ATTRACTION-3, ESCORT, KEYNOTE-181 and ORIENT-2) have shown that PD-1 blockades are associated with a better OS than chemotherapy as second-line therapy for advanced ESCC (15–18). PD-1 blockade can induce the activation of T cells and restore antitumor responses by blocking the binding of PD-1 to its ligand programmed death-ligand 1 (PD-L1) (19). Currently, Nivolumab has been approved by the food and drug administration (FDA) for the treatment of advanced ESCC, regardless of tumor PD-L1 expression levels. Pembrolizumab has also been included as a second-line therapy option for ESCC with PD-L1 expression levels ≥10. Considering the results of ESCORT, camrelizumab has been included in the Chinese Society of Clinical Oncology (CSCO) guidelines for esophageal cancer as the standard second-line treatment for advanced ESCC. Despite the recent advances, the ORR (less than 20%) and PFS (1.7-2.2 months) of immune monotherapy are still far from satisfactory (15–18).

Pre-clinical and clinical studies have shown that anlotinib could modulate the tumor immune microenvironment and increase the infiltration of innate immune cells. When combined with PD-1 blockades, anlotinib conferred considerable synergistic therapeutic benefits. The combination of anlotinib with PD-1 blockade has shown promising activity in hepatocellular cancer(HCC), non-small cell lung cancer (NSCLC) and soft tissue sarcoma in previous clinical studies (20–22). Nevertheless, up to now, there is no relevant literature regarding the combination of anlotinib and PD-1 blockade for patients with advanced ESCC. This retrospective study aims to investigate the efficacy and safety of anlotinib plus PD-1 blockade as second or further-line therapy for advanced ESCC and explore the mechanism of combination therapy to provide sufficient clinical evidence for the subsequent treatment of ESCC.

## Methods

### Patients and study design

**Figure 1:**
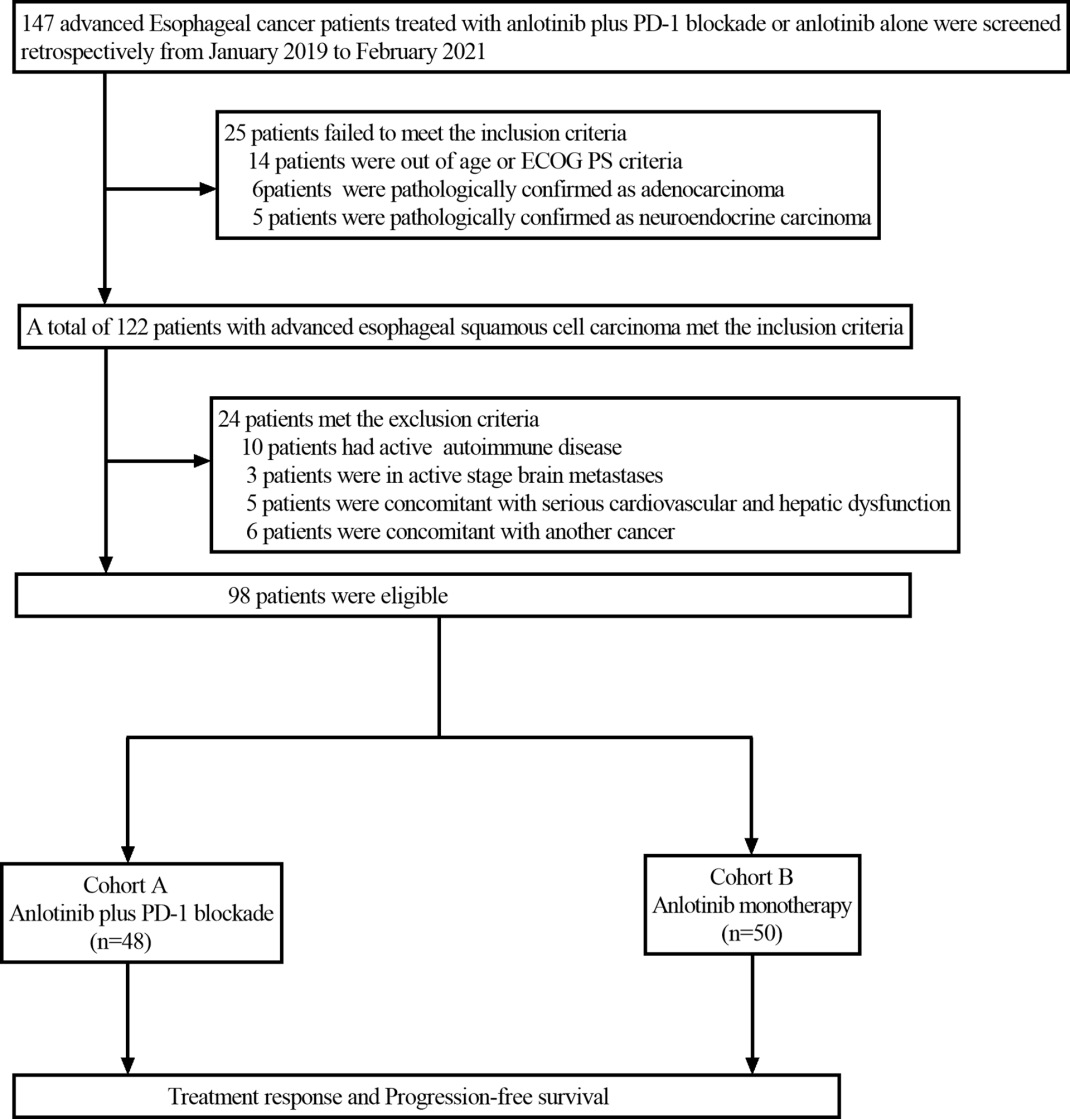
Flow chart of the retrospective study. PD-1 blockade, Programmed death-1 blockade; ECOG PS, Eastern Cooperative Oncology Group Performance status.

This retrospective study reviewed the electronic medical records of patients with esophageal cancer who received anlotinib plus PD-1 blockade or anlotinib monotherapy as second or further-line therapy from January 2019 to February 2021 at Henan Cancer Hospital. The inclusion criteria were as follows: Esophageal squamous cell carcinoma confirmed by histopathology; the patients were aged from 18 to 75 years; disease recurrence or metastasis was confirmed by imaging; Eastern Cooperative Oncology Group Performance Status (ECOG PS)≤2; life expectancy ≥ 3 months; at least one measurable lesion based on the Response Evaluation Criteria in Solid Tumors (RECIST) version 1.1; patients with progressive disease following at least one line of platinum-based combination chemotherapy; adequate bone marrow function (absolute neutrophil count(ANC)≥1.5×10^9^/L, hemoglobin (Hb)≥90g/L, platelets (PLT)≥80×10^9^/L); adequate hepatic and renal function (total bilirubin level ≤ 1.5 times the upper limit of normal (ULN), serum alanine transferase and aspartate aminotransferase ≤ 2.5 times the ULN, serum creatinine ≤ 1.5 times the ULN or calculated creatinine clearance≥60 mL/min based on the standard Cockcroft-Gault formula); adequate cardiac function (left ventricular ejection fraction (LVEF) ≥50%). Patients were excluded if they had active autoimmune diseases, symptomatic brain metastases, uncontrolled hypertension (>150/100 mmHg) despite standard antihypertensive agents, any other malignancy or severe heart, liver and kidney dysfunction. Among the 147 patients screened, 98 patients met the above criteria and were included in the study. Patients with advanced ESCC who received second or further-line treatment of anlotinib plus PD-1 blockade were grouped as cohort A (n=48), and those who received anlotinib monotherapy were grouped as cohort B (n=50). The study profile of the present study is illustrated in **Figure 1**. Patient characteristics of both groups are summarized in **Table 1**. Due to the retrospective nature of the study, informed consent was waived. This study was approved by the Ethics Committee of Henan Cancer Hospital (NO.2020022601).

**Table 1:** Baseline Patient Characteristics.

Characteristic	Anlotinib plus anti-PD-1		Anlotinib		χ2	P-value
	N=48		N=50			
	NO.	%	NO.	%		
Sex						
Male	33	68.8	36	72.0	0.124	0.725
Female	15	31.2	14	28.0
Age,years						
Range	36-75		48-75		/	/
Median	62		63	
ECOG Performance status						
0-1	41	85.4	40	80.0	0.501	0.479
2	7	14.6	10	20.0
History of heavy alcohol use						
Yes	29	60.4	23	46.0	2.044	0.153
No	19	39.6	27	54.0
Smoking history						
Yes	22	45.8	30	60.0	1.973	0.160
No	26	54.2	20	40.0
Location of tumor						
Upper	12	25.0	12	24.0	0.178	0.915
Middle	28	58.3	31	62.0
Lower	8	16.7	7	14.0
Metastases						
Lymph nodes	45	95.8	47	94.0	<0.001	1.000
Lung	15	31.3	19	38.0	0.492	0.483
Liver	13	27.1	17	34.0	0.552	0.458
Bone	10	20.1	9	18.0	0.126	0.723
Metastatic sites						
<2	37	77.1	33	66.0	1.474	0.225
≥2	11	22.9	17	34.0
Number of previous therapy lines						
1 line	28	58.3	33	66.0	0.613	0.434
2 lines or above	20	41.7	17	34.0
Prior radiotherapy						
Yes	29	60.4	25	50.0	1.074	0.300
No	19	39.6	25	50.0
Previous immunotherapy						
Yes	16	33.3	12	24.0	1.045	0.307
No	32	66.7	38	76.0
Primary tumor resection						
Yes	16	33.3	20	40.0	0.468	0.494
No	32	66.7	30	60.0
PD-L1 CPS score						
<1	5	10.4	/	/	/	/
≥1	14	29.2		
<5	7	14.6		
≥5	12	25.0		
<10	9	18.8		
≥10	10	20.8		
Not detected	29	60.4		

PD-L1 CPS score was defined as the number of PD-L1-positive cells (tumor cells, lymphocytes, macrophages) as a proportion of the total number of tumor cells multiplied by 100. ECOG, Eastern Cooperative Oncology Group; Anti PD-1, Anti-programmed death-1; CPS, Combined positive score.

### Treatment plan

A total of 48 patients received anlotinib plus PD-1 blockade. Anlotinib (Chia Tai Tianqing Pharmaceutical, China) was given orally once daily (12 mg) on Days 1–14 of a 21-day cycle. At the same time, the patients were treated with PD-1 blockade. The PD-1 blockades included sintilimab, toripalimab, camrelizumab and pembrolizumab. The 50 patients in cohort B received anlotinib alone. Anlotinib was administered orally once daily at 12mg on Days 1–14 of a 21-day cycle. The dose of anlotinib was adjusted if patients experienced grade 3 or higher serious adverse reactions. All patients received treatment until disease progression, unacceptable toxicity or patient refusal. The detailed medication status of patients is presented in **Table 2**. Blood routine, urine routine, stool routine, liver and kidney function, coagulation function and bone scan were completed before treatment. The pre-treatment assessment included computed tomography (CT)/magnetic resonance imaging (MRI) of the head, chest, abdomen and pelvis. Imaging evaluation was performed for all suspicious lesions. During the treatment period, routine blood, urine, stool and blood biochemistry tests were performed on the patients. The changes of target lesions were evaluated using CT or MRI scans every two cycles or depended on the actual situation when the clinical symptoms of the patients were getting worse.

**Table 2:** The medication status of patients.

Medication group	Medication situation	Number (n)	Medication method
Anlotinib plus PD-1 blockade	Anlotinib plus Toripalimab	11	240mg toripalimab ivgtt D1 +12mg oral anlotinib D1-14 every 3 weeks
	Anlotinib plus Camrelizumab	19	200mg camrelizumab ivgtt D1 + 12mg oral anlotinib D1-14 every 3 weeks
	Anlotinib plus Sintilimab	12	200mg sintilimab ivgtt D1 + 12mg oral anlotinib D1-14 every 3 weeks
	Anlotinib plus Pembrolizumab	6	200mg pembrolizumab ivgtt D1 + 12mg oral anlotinib D1-14 every 3 weeks
Anlotinib	Anlotinib	50	12mg po, D1-14 every 3 weeks

PD-1 blockade, Programmed death-1 blockade.

PD-L1 expression in tumor tissue was evaluated using the 22C3 PD-L1 immunohistochemistry assay (DAKO Autostainer Link48, Agilent Technologies, Carpinteria, CA, USA). PD-L1 expression was reported as a combined positive score (CPS), calculated as the number of PD-L1- positive cells (tumor cells, lymphocytes, and macrophages) divided by the total number of tumor cells, multiplied by 100.

### Evaluation of efficacy and adverse events

Clinical response evaluation was performed with periodic CT or MRI according to Response Evaluation Criteria in Solid Tumors (RECIST 1.1). The clinical efficacy was classified into complete response (CR), partial response (PR), stable disease (SD) and progressive disease (PD). The primary endpoint of this study was the progression-free survival (PFS), defined as the time from treatment initiation to any recorded disease progression, death from any cause, or last follow-up date. Patients lost to follow-up or those who had not progressed at the time of analysis were censored at the time of the last follow-up when we examined PFS. Secondary endpoints included the objective response rate (ORR), disease control rate (DCR) and the incidence of adverse reactions. The ORR was defined as the percentage of patients with a CR or PR. The DCR was defined as the percentage of patients with a CR, PR or SD. Adverse reactions were graded according to the National Cancer Institute Common Toxicity Criteria for Adverse Events version 5.0 (CTCAE 5.0).

### Statistical analysis

The ORR, DCR and incidence rate of toxicity were compared using Chi-square tests or Fisher’s exact tests. Progression-free survival curves were evaluated using the Kaplan–Meier method and compared using the log-rank test. In addition, univariate and COX multivariate regression analysis models were used to investigate the influence of multiple factors on PFS. SPSS 26.0 and GraphPad Prism 8.4 were used for statistical analysis. All statistical tests were two-sided, and the results were considered significant at P<0.05. Follow-up information was collected through outpatient and inpatient medical records or telephone calls. The deadline for follow-up was May1, 2021.

## Results

### Patient characteristics

A total of 98 consecutive patients with advanced ESCC were enrolled between January 2019 and February 2021. Of the 98 patients selected, including 69 (70.4%) males and 29 (29.6%) females. 48 patients received anlotinib plus PD-1 blockade (cohort A), and 50 patients accepted anlotinib monotherapy (cohort B). The median age was 62 years (36-75 years) in cohort A and 63 years (48-75 years) in cohort B. In cohort A, 16 patients underwent primary tumor resection, while 20 did so in cohort B. A total of 92 patients developed lymph node metastasis, including 45 patients in cohort A and 47 patients in cohort B. Moreover, 72 patients developed distant organ metastasis, including 32 patients in cohort A and 40 patients in cohort B. The two groups were similar in other characteristics including gender, ECOG score and histological type, etc.

### Response and survival

**Figure 2:**
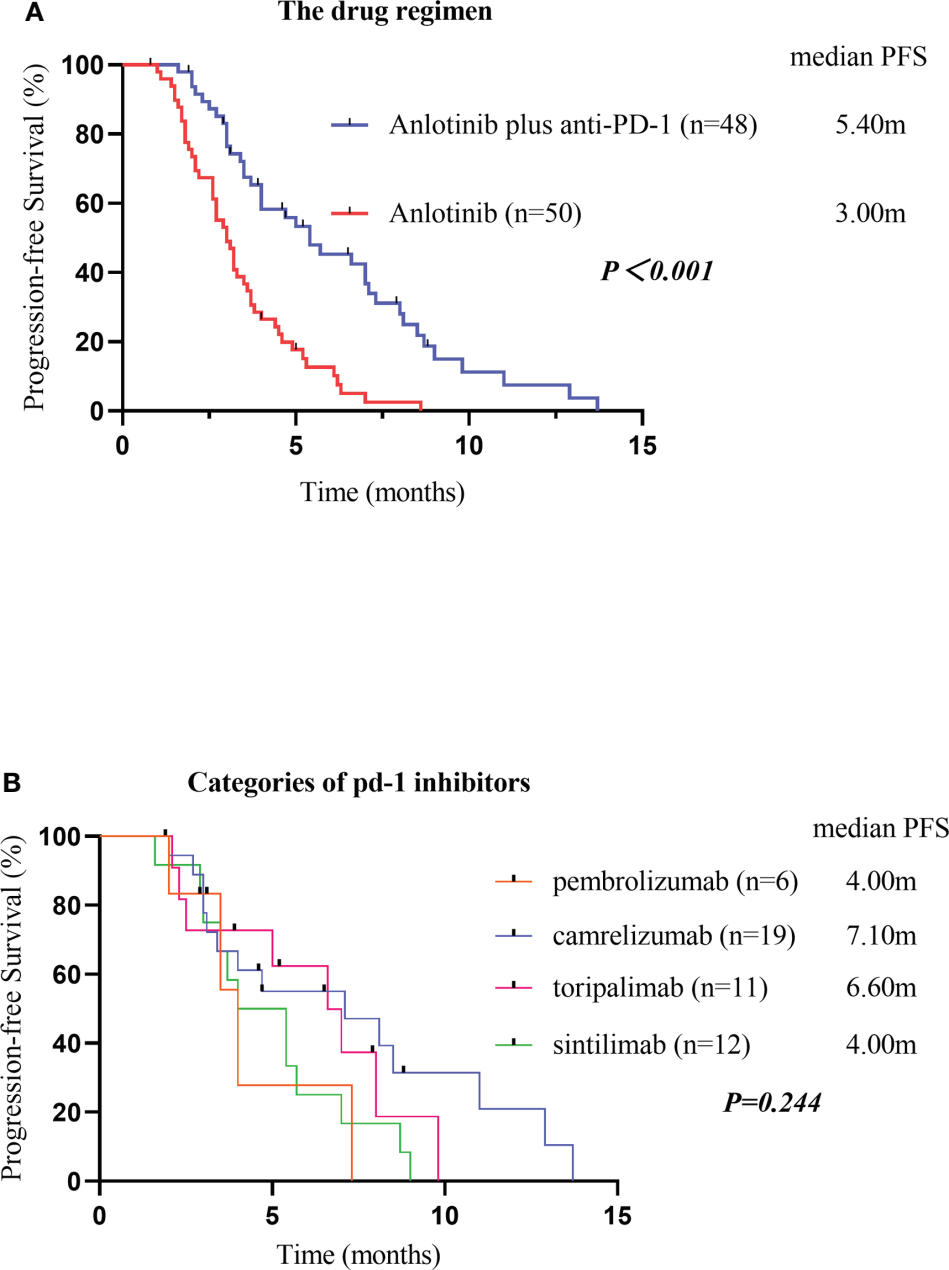
Kaplan-Meier plot curves for the PFS of patients in different groups. Compared with anlotinib alone, anlotinib plus PD-1 blockade showed higher mPFS (P<0.001) **(A)**. PFS, Progression-Free Survival; PD-1 blockade, Programmed death-1 blockade. PFS of different categories of PD-1 blockades **(B)**. PFS, Progression-Free Survival; PD-1 blockades, Programmed death-1 blockades.

**Figure 3:**
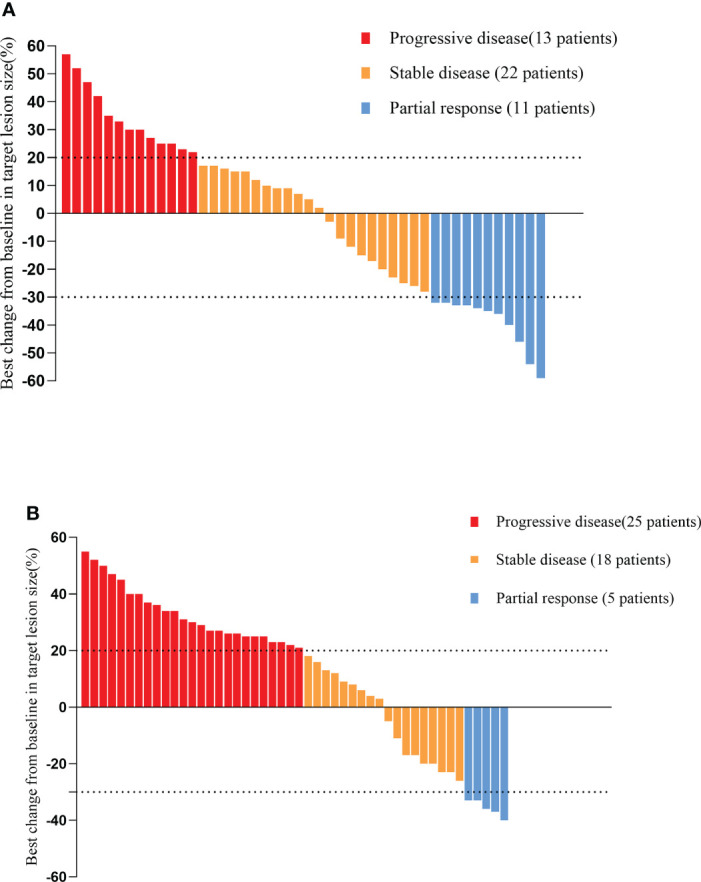
Waterfall plot illustrating maximum change in target lesion size based on the tumor response in patients treated with anlotinib plus PD-1 blockade **(A)**/anlotinib alone **(B)**. PD-1 blockade, Programmed death-1 blockade.

**Figure 4:**
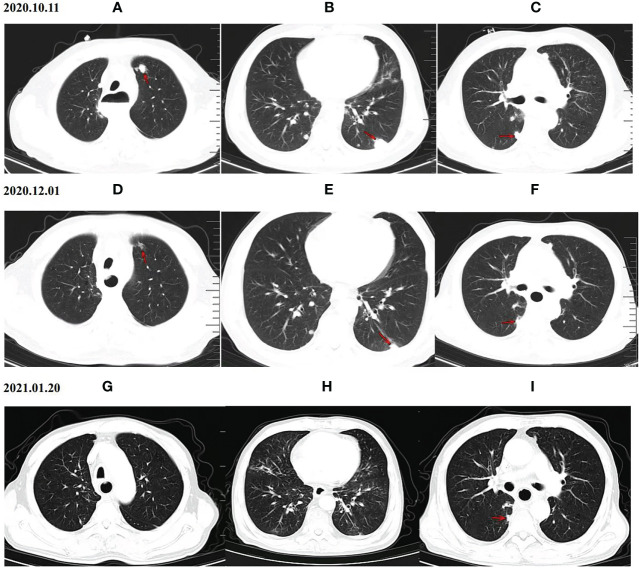
CT scan results of the changes for target lesions in one patient with esophageal squamous cell carcinoma before and after anlotinib combined with PD-1 blockade administration. Upper panel **(A–C)** CT scan of the patient’s lung before the treatment (October 11th, 2020), Middle panel **(D–F)** CT scan of the patient’s lung after 2 cycles of treatment with anlotinib plus PD-1 blockade (December 1th, 2020), PR was achieved after 2 cycles of treatment. Lower panel **(G–I)** Chest CT images after 4 cycles of treatment with anlotinib plus PD-1 blockade (January 20th, 2021), Chest CT on January 20th, 2021 indicated a significant shrink in pulmonary metastasis. PD-1 blockade = Programmed death-1 blockade.

As of the data cutoff on May 1, 2021, the median follow-up time was 9.30 months (8.23–10.37 months) in cohort A and11.10 months (7.82–14.38 months) in cohort B. Four (4.1%) patients were lost to follow-up, including two (4.2%) patients from cohort A and two (4%) patients from cohort B. Disease progression was observed in 38 patients (79.2%) in cohort A and 47 patients (94.0%) in cohort B at the time of data cutoff. A significantly longer mPFS was achieved in cohort A compared to cohort B (5.40 months vs. 3.00 months, P < 0.001) (**Figure 2A**). Furthermore, the survival with different PD-1 blockade were analyzed, indicating no significant difference in PFS between different types of PD-1 blockade (P=0.244, **Figure 2B**). At data cutoff, 13 deaths (27.1%) were recorded in cohort A and 16 deaths (32%) in cohort B. The median OS for the two cohorts was not reached. A total of 94 patients were evaluable for response, including 46 patients of anlotinib plus PD-1 blockade and 48 patients of anlotinib monotherapy. The clinical responses were as follows: CR was not observed in both groups, 11patients (23.9%) achieved PR in cohort A, while 5 patients (10.4%) did so in cohort B. The ORR was 23.9% and 10.4% for cohorts A and B, respectively (P=0.082). The DCR of cohort A (71.7%) in advanced ESCC was higher than that of cohort B (47.9%) (P=0.019). The ORR and DCR among patients with measurable disease are summarized in **Table 3**. For each patient, the percent change in the sum of the longest diameter of target lesions diameter from the baseline was graphed in a waterfall plot (**Figures 3A, B**). In addition, the CT images of an esophageal squamous cell carcinoma patient with lung metastasis whose tumor continued to shrink after treatment with anlotinib plus PD-1 blockade were recorded (**Figure 4**).

**Table 3:** Analysis of efficacy.

Best overall response	Anlotinib plus PD-1 blockade			Anlotinib		P-value
	N=46			N=48		
	NO.	％		NO.	％	
Complete response (CR)	0	0		0	0	
Partial response (PR)	11	23.9		5	10.4	
Stable disease (SD)	22	47.8		18	37.5	
Progression disease (PD)	13	28.3		25	52.1	
Overall response rate (ORR)	11	23.9		5	10.4	0.082
Disease control rate (DCR)	33	71.7		23	47.9	0.019*

Response was assessed according to Response Evaluation Criteria in Solid Tumors (RECIST version 1.1). PD-1 blockade, Programmed death-1 blockade; * indicates p value less than 0.05

As of the data cutoff on May 1, 2021, 4 patients did not undergo efficacy evaluation due to loss to follow-up.

### Toxicity

In the present study, the adverse reactions across the two groups were mainly evaluated as grade 1 or 2. The common grade 1-2 hematologic toxicities included anemia (18.8% in cohort A, 14% in cohort B, P=0.525), leucopenia (14.6% in cohort A, 16% in cohort B, P=0.846) and thrombocytopenia (20.8% in cohort A, 18% in cohort B, P=0.723). The mainly grade 1/2 non-hematologic toxicities included hypertension (52.1% in cohort A, 54% in cohort B, P=0.849), appetite loss (41.7% in cohort A, 38.00% in cohort B, P=0.711), hypothyroidism (39.6% in cohort A, 20.8% in cohort B, P=0.034), hyperlipidemia (37.5% in cohort A, 34.00% in cohort B, P=0.718), fatigue (33.3% in cohort A, 30% in cohort B, P=0.723) and diarrhea (29.2% in cohort A,22% in cohort B, P=0.416). Three patients (6.3%) developed grade 1/2 immune-related pneumonia in cohort A. The rates of serious 3-4 grade adverse events were similar between the two groups (20.8% in cohort A,14% in cohort B, P=0.372). The key grade 3/4 toxicities in cohort A were hypertension, hand–foot syndrome, fatigue, hyperlipidemia, diarrhea and elevated transaminase. Dose reduction was required in nine patients (18.8%) due to treatment-related adverse reactions in cohort A, and six patients (12.0%) in cohort B (**Table 4**). Three patients (6.3%) discontinued treatment due to immune-related pneumonia in cohort A. In cohort B, there were no patients discontinued treatment due to the toxic effects. Toxicities associated with treatment were clinically manageable. No treatment-related deaths occurred. Details of the toxicities are listed in **Table 5**.

**Table 4:** Treatment administration and dose modification of anlotinib.

Dose of anlotinib (mg)	Number (n)	Number(n)
	Anlotinib plus PD-1 blockade	Anlotinib
Initial dosage (mg)		
12	48	50
Modification of dosage (mg)		
12 → 10	9	6

PD-1 blockade, Programmed death-1 blockade.

**Table 5:** Treatment-related adverse events in the two groups.

	Grade 1-2,N (%)			Grade 3-4, N (%)		
Adverse Events	Anlotinib plus PD-1 blockade (N=48)	Anlotinib (N=50)	P-value	Anlotinib plus PD-1 blockade (N=48)	Anlotinib (N=50)	P-value
Hematologic						
Leucopenia	7(14.6%)	8(16.0%)	0.846	0	0	0
Anemia	9(18.8%)	7(14.0%)	0.525	0	0	0
Thrombocytopenia	10(20.8%)	9(18.0%)	0.723	0	0	0
Gastrointestinal						
Nausea/Vomiting	10(20.8%)	8(16.0%)	0.537	0	1(2.0%)	1.000
Diarrhea	14(29.2%)	11(22.0%)	0.416	1(2.1%)	0	0.490
Appetite loss	20(41.7%)	19(38.0%)	0.711	0	0	0
Abdominal pain/Abdominal distension	8(16.7%)	7(14.0%)	0.714	0	0	0
Constipate	6(12.5%)	4(8.0%)	0.688	0	0	0
Liver function						
Elevated transaminase	11(22.9%)	9(18.0%)	0.546	1(2.1%)	0	0.490
Hyperbilirubinemia	12(25%)	8(16.0%)	0.269	0	0	0
Elevated lactate dehydrogenase	8(16.7%)	6(12.0%)	0.509	0	0	0
Others						
Hypothyroidism	19(39.6%)	10(20.0%)	0.034*	0	0	0
Fatigue	16(33.3%)	15(30.0%)	0.723	2(4.2%)	1(2.0%)	0.971
Hypoproteinemia	12(25%)	11(22.0%)	0.726	0	0	0
Hypertension	25(52.1%)	27(54.0%)	0.849	3(6.3%)	2(4.0%)	0.963
Hand–foot syndrome	9(18.8%)	7(14.0%)	0.525	2(4.2%)	1(2.0%)	0.971
Bleeding	4(8.3%)	5(10.0%)	1.000	0	0	0
Hyperlipidemia	18(37.5%)	17(34.0%)	0.718	1(2.1%)	1(2.0%)	1.000
Hyponatremia	6(12.5%)	6(12.0%)	0.940	0	1(2.0%)	1.000
Immune-related pneumonia	3(6.3%)	0	0.227	0	0	0
Cough/Expectoration	9(18.8%)	5(10.0%)	0.216	0	0	0
Hypokalemia	3(6.3%)	2(4.0%)	0.963	0	0	0
Hypocalcemia	5(10.4%)	3(6.0%)	0.668	0	0	0
pruritus	7(14.6%)	2(4.0%)	0.143	0	0	0
Proteinuria	7(14.6%)	10(20.0%)	0.479	0	0	0

PD-1 blockade, Programmed death-1 blockade; * indicates p value less than 0.05.

### PD-L1 expression

**Figure 5:**
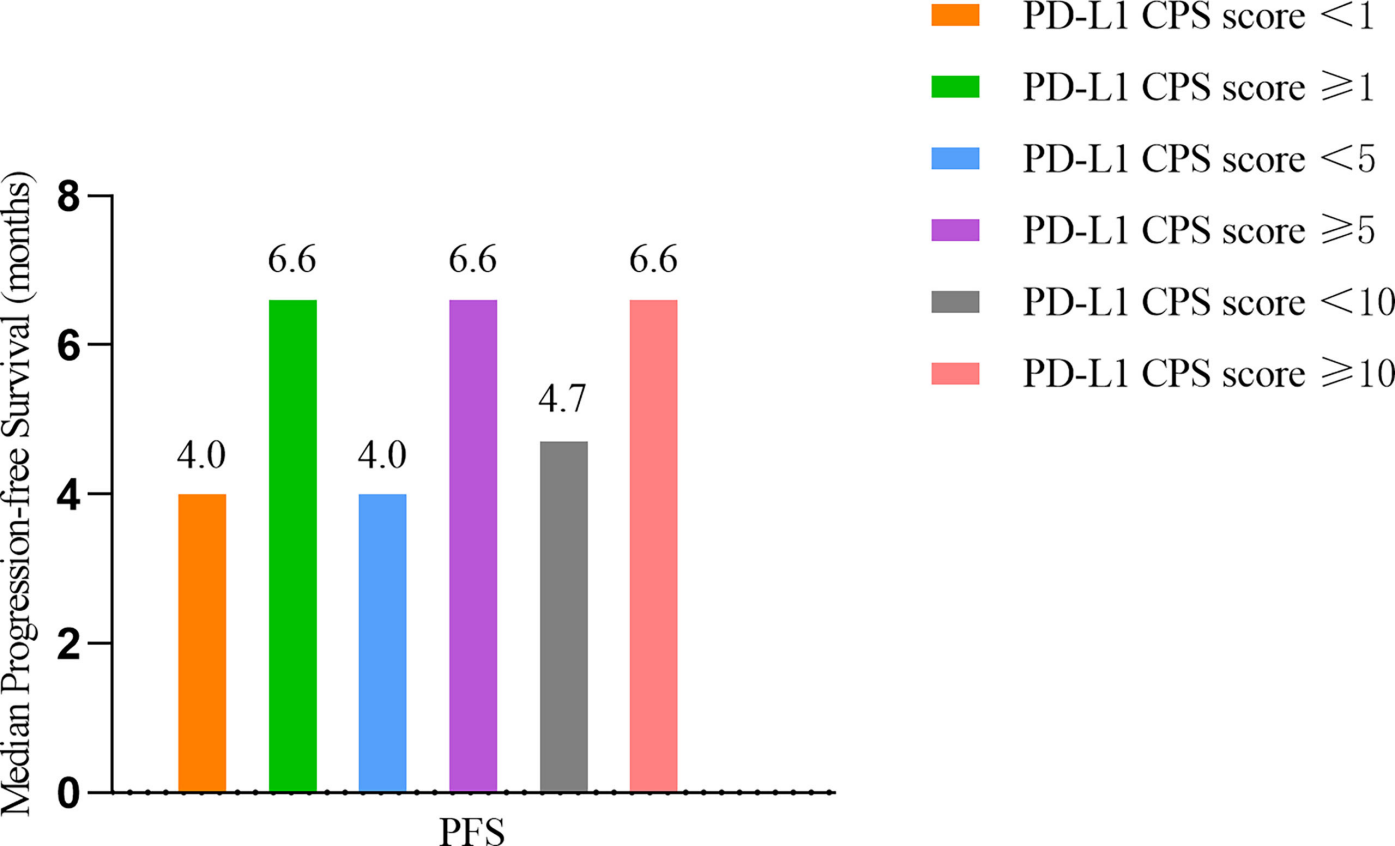
Survival outcomes of different PD-L1 CPS score. PD-L1 CPS= Programmed death- Ligand 1 Combined positive score.

Evaluable tissue samples were collected from 19 patients to assess baseline PD-L1 expression. Among them, 14 (73.7%) had a PD-L1 CPS of ≥1, and 10 (52.6%) had a PD-L1 CPS of ≥10. In the patients with a PD-L1 CPS of ≥1, the ORR was 28.6% (4/14), and the DCR was 71.4% (10/14). The ORR and DCR were 30.0% (3/10) and 70.0% (7/10) in the patients with a PD-L1 CPS of ≥10 (**Table 6**). The median PFS for patients with a PD-L1 CPS of ≥10 was 6.60 months (95% CI: 1.95 -11.25months), whereas the median PFS for those with a PD-L1 CPS of < 10 was 4.70months (95%CI: 2.53-6.87months) (**Figure 5**).

**Table 6:** Responses by PD-L1 Expression.

	N	CR	PR		SD	PD	Objective responses		Disease control		
PD-L1 combined positive score											
<1	5	0		1	2	2	1/5(20%)	P=1.000		3/5(60%)	P=1.000
≥1	14	0		4	6	4	4/14(28.6%)			10/14(71.4%)	
<5	7	0		1	3	3	1/7(14.3%)	P=0.603		4/7(57.1%)	P=0.617
≥5	12	0		4	5	3	4/12(33.3%)			9/12(75%)
<10	9	0		2	4	3	2/9(22.2%)	P=1.000		6/9(66.7%)	P=1.000
≥10	10	0		3	4	3	3/10(30%)			7/10(70%)

Responses were assessed according to Response Evaluation Criteria in Solid Tumors (RECIST version 1.1).

PD-L1, programmed death-ligand 1; CR, Complete response; PR, Partial response; SD, Stable disease; PD, Progressive disease.

### Univariate analysis

Univariate analysis revealed that sex, age, smoking history, drinking history, prior radiotherapy, previous immunotherapy, primary tumor resection, tumor location and lymph node metastasis had no influence on the median PFS (P >0.05). However, ECOG PS, the drug regimen, distant organ metastasis and metastatic sites were reliable prognostic factors. In addition, with distant organ metastasis (P=0.001) and metastatic sites≥ 2 (P=0.001) were negative factors for mPFS, while ECOG PS ≤ 1 was identified as a positive factor (P=0.006). The results are given in **Table 7**.

**Table 7:** Univariate analysis of PFS of 98 advanced esophageal squamous cell carcinoma patients.

Characteristic	No. of Patients	HR	95% CI	P-value
Sex		0.816	0.508-1.313	0.403
Male	69			
Female	29			
Age		1.117	0.711-1.757	0.631
<65	61			
≥65	37			
ECOG PS		2.207	1.260-3.866	0.006*
0-1	81			
2	17			
History of heavy alcohol use		1.052	0.681-1.626	0.820
Yes	52			
No	46			
Smoking history		1.241	0.802-1.922	0.332
Yes	52			
No	46			
Location of tumor		/	/	0.396
Upper	24			
Middle	59			
Lower	15			
Lymph node metastasis		1.438	0.623-3.320	0.394
Yes	92			
No	6			
Distant organ metastasis		2.457	1.430-4.220	0.001*
Yes	72			
No	26			
Metastatic sites		2.186	1.364-3.504	0.001*
<2	70			
≥2	28			
Primary tumor resection		0.667	0.423-1.051	0.081
Yes	36			
No	62			
Prior radiotherapy		1.060	0.686-1.639	0.792
Yes	54			
No	44			
Previous immunotherapy		0.722	0.439-1.189	0.201
Yes	28			
No	70			
Number of previous therapy lines		1.556	0.994-2.437	0.053
1 line	61			
2 lines or above	37			
The drug regimen		3.004	1.871-4.823	<0.001*
Anlotinib plus PD-1 blockade	48			
Anlotinib monotherapy	50			

ECOG PS, Eastern Cooperative Oncology Group Performance Status; PD-1, programmed death-1; HR, Hazard Ratio; CI, Confidence Interval; * indicates p value less than 0.05.

### Multivariate cox regression analysis

**Figure 6:**
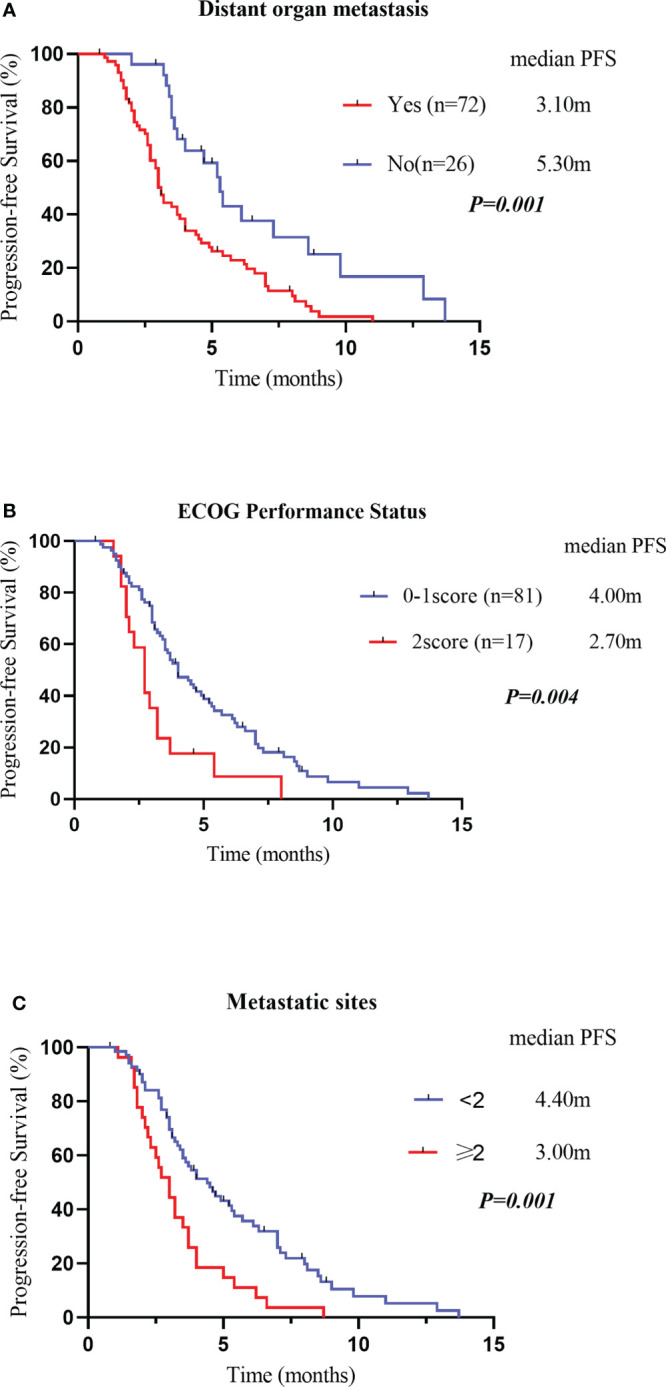
Kaplan–Meier survival curves for PFS were compared among patients with or without distant organ metastasis **(A)**. PFS, Progression-Free Survival Kaplan–Meier survival curves for PFS were compared among patients with different Eastern Cooperative Oncology Group performance statuses **(B)**. PFS, Progression-Free Survival Kaplan–Meier survival curves for PFS were compared among patients with different metastatic sites **(C)**. PFS, Progression-Free Survival.

**Figure 7:**
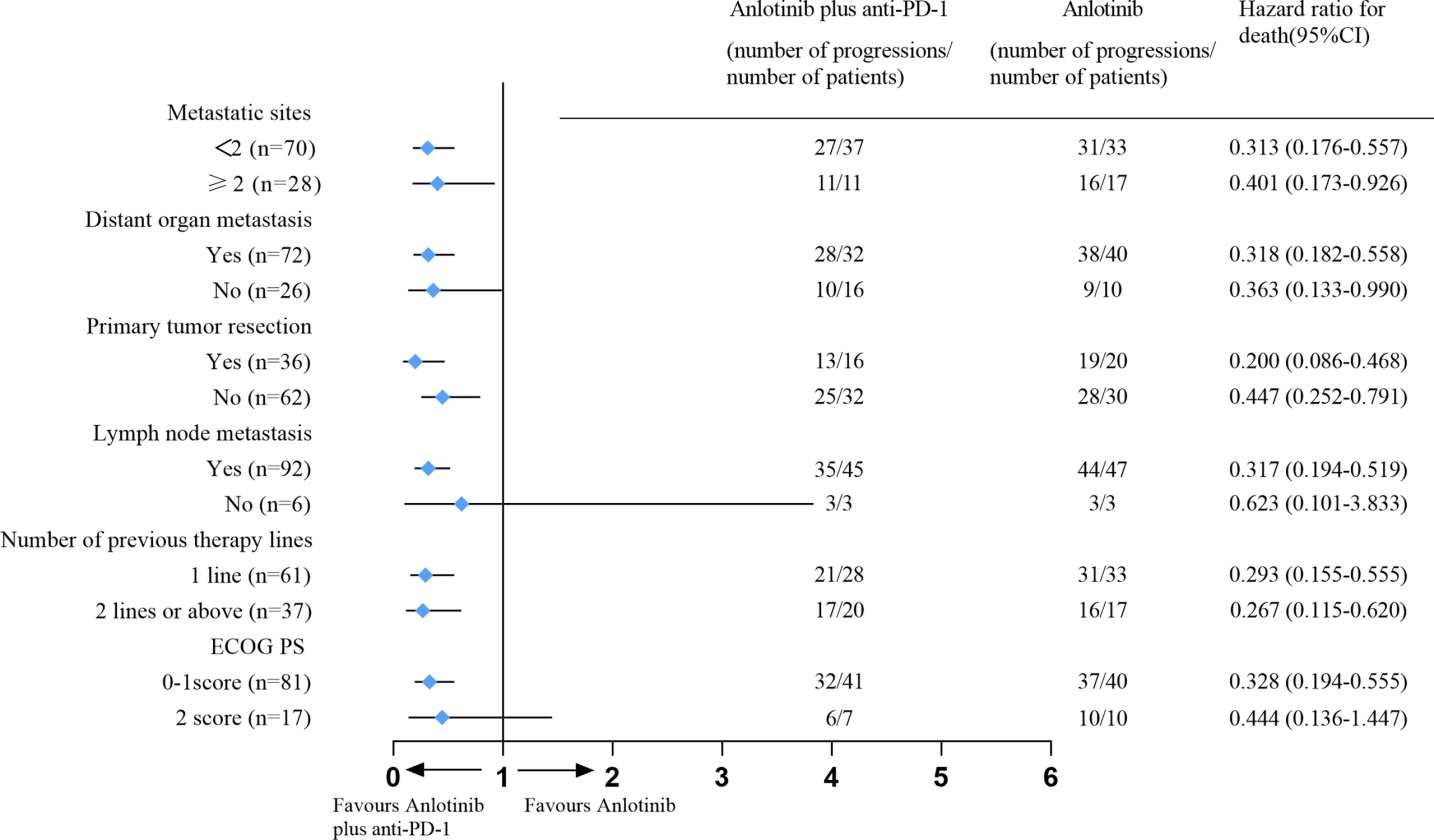
Forest plot for subgroup analysis of PFS. Hazard ratios and the corresponding 95% CIs for anlotinib plus PD-1 blockade relative to anlotinib monotherapy were calculated using the Cox proportional hazards mode. PFS, Progression-Free Survival; PD-1blockade, Programmed death-1 blockade; ECOG PS, Eastern Cooperative Oncology Group Performance status.

Variables that were statistically significant (P<0.05) in the univariate analysis were subsequently entered into a multivariate Cox regression model. The results indicated that the drug regimen, ECOG PS, distant organ metastasis and metastatic sites were independent factors affecting PFS in patients with advanced ESCC, as shown in **Table 8**. As displayed in **Figures 6A–C**, the median PFS of patients without distant organ metastasis was 5.30 months, and the median PFS of patients with distant organ metastasis was 3.10 months (P=0.001). Patients with an ECOG PS of 0–1 had a significantly higher median PFS than patients with an ECOG PS of 2 (4.00vs. 2.70 months, P=0.004). Moreover, patients with less than two metastatic sites had a higher PFS than those with two or more metastatic sites (4.40 vs. 3.00 months, p=0.001). In all the tested subgroups, longer PFS was achieved with anlotinib plus PD-1 blockade compared to anlotinib monotherapy (**Figure 7**).

**Table 8:** Multivariate Cox regression analysis of PFS of 98 advanced esophageal squamous cell carcinoma patients.

Characteristic	HR	95% CI	P-value
ECOG PS (2 vs. 0–1)	2.533	1.423-4.509	0.002*
Distant organ metastasis (Yes vs. NO)	2.197	1.231-3.923	0.008*
Metastatic sites (≥2 vs.<2)	1.739	1.050-2.882	0.032*
The drug regimen (Anlotinib monotherapy vs. Anlotinib plus PD-1 blockade)	3.114	1.909-5.080	<0.001*

ECOG PS, Eastern Cooperative Oncology Group Performance Status; PD-1, Programmed death-1; HR, Hazard Ratio; CI, Confidence Interval; * indicates p value less than 0.05.

## Discussion

Due to the aggressive nature of the disease and limited effective treatment options, the clinical management of advanced ESCC remains challenging. Immune checkpoint inhibitor monotherapy as second or third-line treatment has shown limited therapeutic benefit in patients with ESCC. In the KEYNOTE-181, ATTRACTION-3, ESCORT and ORIENT-2 studies, the ORRs of PD-1 blockade monotherapy were 16.7% (median PFS of 2.2 months), 19.0% (median PFS of 1.7 months), 20.2% (median PFS of 1.9 months) and 12.6% (median PFS of 1.6months), respectively (15–18). Our results demonstrated the superior efficacy of anlotinib plus PD-1 blockade in the second-line setting, as shown by the ORR of 23.9% and median PFS of 5.40 months.

In our retrospective study, the second or further-line treatment of anlotinib monotherapy in advanced ESCC resulted in a median PFS of 3.00 months and a DCR of 47.9%, In contrast, the combination treatment showed a median PFS of 5.40 months and a DCR of 71.7%, presenting an additional 2.40 months PFS and 23.8% DCR benefit compared with anlotinib (P<0.05). No statistically significant difference was observed in ORR between the two groups. The combination of anlotinib with PD-1 blockade displayed an ORR of 23.9%, and patients in the anlotinib group had an ORR of 10.4% (P=0.082). In summary, treatment with anlotinib plus PD-1 blockade generated longer PFS, better DCR and ORR than anlotinib monotherapy in previously treated patients with advanced ESCC. The above phenomena may be associated to the following reasons. Anlotinib combined with PD-1 blockade may produce synergistic effects, which can promote vascular remodeling, improve tumor immune microenvironment, increase the activation of immune effector cells and further improve anti-tumor efficacy. 1) Mature dendritic cells (DC) play an essential role in immunotherapy, and VEGF inhibits the differentiation and maturation of DC by binding to VEGFR-2. Due to the lack of expression of major histocompatibility complex molecules (MHC-1, MHC-2) and costimulatory molecules (B7-1, B7-2), immature DCs cannot present tumor antigens to T cells. Anlotinib can reverse the inhibitory effects of VEGF on dendritic cells, leading to the initiation and activation of T cells. 2) Anlotinib normalizes the tumor vasculature and promotes the infiltration of effector T cells into the tumor. 3) Anlotinib can inhibit the activity of myeloid-derived suppressor cells (MDSCs) and regulatory T cells (Tregs), and polarize tumor-associated macrophages (TAMS) from the immune-inhibitory M2-like phenotype toward the immune-stimulatory M1-like phenotype. Eventually, the immunosuppressive tumor microenvironment is successfully reprogrammed into an immunostimulatory microenvironment. 4) PD-1 blockades reinforce the ability of T cells to attack tumor cells and improve vessel normalization by indirectly down-regulating the angiogenic factors (23, 24).

The adverse reactions in the present study were mainly graded 1-2 in both groups. Still, grade 1–2 hypothyroidism of patients in the anlotinib plus PD-1 blockade group was significantly more common than in the anlotinib monotherapy group (P=0.034). PD-1 blockades can enhance anti-tumor responses and promote the body’s normal immune function, resulting in immune imbalance and a series of immune-related adverse reactions. In our study, grade1-2 immune-related pneumonia occurred in 3 patients (6.3%), and the symptoms and radiographic abnormalities finally subsided with corticosteroid therapy. Univariate and multivariate Cox regression analyses showed that the drug regimen, ECOG PS, distant organ metastasis and metastatic sites were independent factors affecting PFS in patients with advanced ESCC (P<0.05).

In the current study, poorer outcomes were observed in the anlotinib monotherapy group compared to the findings of the ALTER-1102 trial, which also assessed the efficacy and safety of anlotinib as a second or further-line treatment in advanced ESCC. The results of the ALTER-1102 trial indicated that the DCR (64.0% vs. 18.0%, P < 0.001) and median PFS (3.02 vs. 1.41 months, P < 0.001) were higher in the anlotinib group than in the placebo group (14). In the present study, the DCR (47.9%) and PFS (3.0 months) of the anlotinib group were shorter than those in the ALTER-1102 study. This discrepancy may partially arise from the retrospective design of our study, as the clinical trial might have benefited from better patient adherence. Furthermore, the patients’ physical conditions and drug responses might be different between ALTER-1102 and our study.

Previous studies have analyzed the interrelation between anti-angiogenic dose and efficacy, reporting that low-dose anti-VEGF/VEGFR monoclonal antibodies/VEGFR-2 TKIs are contributed to normalizing the tumor vasculature and inhibiting the growth of new vessels. High-dose anti-VEGF/VEGFR monoclonal antibodies/VEGFR-2 TKIs may excessively prune the vasculature, causing severe hypoxia and immunosuppression. Lower doses are superior to high doses for inducing a relatively immune-supportive tumor microenvironment (25). In contrast, one study on lung cancer animal models suggested that high-dose anlotinib plus PD-1 blockade caused significantly greater tumor inhibition than low-dose anlotinib. Anlotinib increased infiltration of the innate immune cells, including natural killer cells and antigen-presenting cells, whereas the percentage of M2-like tumor-associated macrophages was significantly reduced. These immune-stimulatory characteristics seemed to correlate positively with the anlotinib dose. This might be because anlotinib is a multi-targeted tyrosine kinase inhibitor; besides VEGFR1-3, it also targets FGFR1-4, PDGERα/β, c-KIT and MET (26). In the present study, the dose of anlotinib was 12mg/d in both groups. Investigating the optimal dosage and administration sequence of anlotinib and PD-1 blockade could be a direction for future research.

In order to better select the population who will achieve optimal benefit, accurate biomarkers are required to predict the efficacy of treatment in ESCC. Tumor mutation burden (TMB) is a potential predictive immunotherapy biomarker in multiple solid tumors. However, the role of TMB as an independent predictor of esophageal cancer immunotherapy efficacy remains controversial (27). Mismatched repair protein deficiency (dMMR) and microsatellite instability-high (MSI-H) are predictors of the efficacy of anti-PD-1 monoclonal antibodies. Unfortunately, dMMR and MSI-H occur in 5% or less of Chinese patients with ESCC (28). PD-L1 expression is deemed a predictor of immunotherapy response in many types of cancers, such as lung cancer and gastric cancer (29). In the ATTRACTION-3 and ESCORT-3 trials, the OS benefit of nivolumab or camrelizumab compared with chemotherapy was found irrelevant to PD-LI expression in the second-line therapy of ESCC. However, the KEYNOTE-181 trial indicated that patients with PD-L1 combination positive score (CPS)≥10 had a longer OS compared with chemotherapy for ESCC (15–17). In the present study, we noted that the median PFS for patients with PD-L1 CPS of ≥10 was longer than those with PD-L1 CPS < 10. The ORR and DCR for patients with PD-L1 CPS ≥ 1 and ≥10 were similar. The interpretation of these results is difficult due to the relatively small sample size of our study and the complex interactions between tumor and immune cells in the tumor immune microenvironment. Therefore, the predictive value of PD-L1 expression in esophageal cancer still needs further investigation.

This study had several limitations. First, it was a retrospective study with small sample size. The therapeutic activity of anlotinib plus PD-1 blockade still needs to be assessed in more patients. In addition, due to the small sample size, propensity score matching and stratified analysis were not used, so it is necessary to note that bias may exist when quoting the results of this study. Second, the TMB and MMR status was not evaluated. Third, the follow-up duration was relatively short, and OS was not reached. However, the results in our study were considered meaningful due to the limited literature on similar prospective studies. Further prospective randomized studies will be required to provide more conclusive evidence.

## Conclusion

In conclusion, anlotinib plus PD-1 blockade revealed encouraging survival outcomes among patients with advanced ESCC, with a tolerable toxicity profile. The combination of anlotinib with PD-1 blockade could serve as a new therapeutic option for patients with advanced ESCC.

## Data availability statement

The original contributions presented in the study are included in the article/Supplementary Material. Further inquiries can be directed to the corresponding author.

## Ethics statement

The studies involving human participants were reviewed and approved by the ethics committee of affiliated cancer hospital of zhengzhou university. The ethics committee waived the requirement of written informed consent for participation.

## Author contributions

YL (1st author) contributed to the conception and design of the research. YL (1st author) and QG contributed to the acquisition of the data. YL (1st author) and SX contributed to the analysis and interpretation of the data. SX and KL contributed to the statistical analysis. YL (1st author) contributed to the drafting of the manuscript. YL (the corresponding author) contributed to the revision of the manuscript. All authors contributed to the article and approved the submitted version.

## Funding

This research was supported by Beijing Kangmeng Charity Foundation Medical Research and Development Fund-Clinical and Basic Research Project (TB204036).

## Acknowledgments

We appreciate the patients who participated in this study. We also thank our colleges for their devoted support.

## Conflict of interest

The authors declare that the research was conducted in the absence of any commercial or financial relationships that could be construed as a potential conflict of interest.

## Publisher’s note

All claims expressed in this article are solely those of the authors and do not necessarily represent those of their affiliated organizations, or those of the publisher, the editors and the reviewers. Any product that may be evaluated in this article, or claim that may be made by its manufacturer, is not guaranteed or endorsed by the publisher.

## Glossary

**Table:**

Anti-PD-1	Anti-Programmed Death-1
CPS	Combination Positive Score
CT	Computed tomography
CR	Complete Response
CTCAE	Common Toxicity Criteria for Adverse Events
CSCO	Chinese Society of Clinical Oncology
DC	Dendritic Cells
dMMR	Mismatched Repair Protein Deficiency
DCR	Disease Control Rate
ECOG	Eastern Cooperative Oncology Group
EC	Esophageal cancer
ESCC	Esophageal Squamous Cell Carcinoma
EAC	Esophageal adenocarcinoma
FGFR	Fibroblast Growth Factor Receptor
FDA	Food and Drug Administration
HCC	Hepatocellular carcinoma
MRI	Magnetic resonance imaging
MHC	Major histocompatibility complex molecules
MDSCs	Myeloid-derived suppressor cells
MSI-H	Microsatellite instability-high
NSCLC	Non-Small Cell Lung Cancer
OS	Overall Survival
ORR	Objective Response Rate
PDGFR	Platelet-Derived Growth Factor Receptor
PD-1	Programmed death-1
PD-L1	Programmed death ligand-1
PR	Partial Response
PD	Progressive Disease
PFS	Progression-Free Survival
RECIST	Response Evaluation Criteria in Solid Tumors
SD	Stable Disease
TKI	Tyrosine Kinase Inhibitor
Tregs	Regulatory T cells
TAMs	Tumor-associated macrophages
TMB	Tumor mutation burden
VEGF	Vascular Endothelial Growth Factor
VEGFR	Vascular Endothelial Growth Factor Receptor.
